# Implanting Teeth

**Published:** 1888-07

**Authors:** 


### IMPLANTING TEETH.

It is claimed that Dr. Yonger, of San Francisco, was the first dentist in this country to perform successfully the experiment of implanting teeth. This process is not to be confounded with transplanting teeth, which has been practised by dentists for many years. In the latter operation, a tooth freshly extracted is inserted in a socket from which one has just been drawn, and the parts unite, circulation between the jaw and the tooth is established, and the latter actually takes the place of its predecessor.

In Dr. Yonger's experiment the tooth to be replaced has long been extracted, and the socket filled up with bony substance. He drills into the jaw, gouges out a new socket, and then, taking a tooth that has long been extracted, he cleans it thoroughly, soaks it in bichloride of mercury, and inserts it in the socket just formed. This new tooth in due time becomes firmly anchored, and as serviceable as the original one before it became decayed. Dr. Younger holds that the tooth is held in its place by the soft tissues surrounding it, and that the artificial socket has nothing to do with anchoring it.

The experiment described above was performed by Dr. G. M. Curtis, of Syracuse, N. Y., who afterward extracted the implanted tooth, and sent it to Dr. W. M. Gray, the microscopist of the Surgeon-General's Office, who has made a very careful examination of it. His experiments proved beyond question that the tooth so implanted is revived, the circulation is established between the socket and the implanted tooth, and that the socket does take an active part in anchoring the tooth. A tooth so implanted is much more firmly anchored in the jaw than one of the originals, and, in the case referred to, the tooth was held so firmly that Dr. Curtis broke it in extracting it. Dr. Gray does not doubt that the soft tissues do take an active part in the operation, but he has proved his propositions in regard to the bone and the tooth beyond all question.

One of the staff of HALL'S JOURNAL OF HEALTH has given some attention to this subject as an observer cf the new process of restoring lost teeth. The operation was performed upon patients at the dental rooms of Dr. J. Albert Kimball, of New York City, by his chief assistant, Dr. Attolingua. The root cavities occupied by the decayed teeth were deepened, and natural teeth from other mouths, which had been extracted a considerable length of time, inserted. In one instance an inward-growing sound tooth was extracted, a new root cavity bored and the same tooth inserted in an upright position.

So skilfully indeed were these operations performed, that dentists who were permitted to examine the work after the lapse of a few days, failed to detect the newly installed member. We regard the achievement in dental surgery which dispenses with the objectionable plates and pivots, and replaces the lost members with step-children that good mother nature takes to so kindly, as the *ne plus ultra* of dentistry.

